# Leveraging Genetic Data to Elucidate the Relationship Between COVID‐19 and Ischemic Stroke

**DOI:** 10.1161/JAHA.121.022433

**Published:** 2021-11-10

**Authors:** Verena Zuber, Alan Cameron, Evangelos P. Myserlis, Leonardo Bottolo, Israel Fernandez‐Cadenas, Stephen Burgess, Christopher D. Anderson, Jesse Dawson, Dipender Gill

**Affiliations:** ^1^ Department of Epidemiology and Biostatistics School of Public Health Imperial College London London UK; ^2^ Dementia Research Institute at Imperial College London London UK; ^3^ Institute of Cardiovascular and Medical Sciences University of Glasgow UK; ^4^ Center for Genomic Medicine Massachusetts General Hospital Boston MA; ^5^ McCance Center for Brain Health Massachusetts General Hospital Boston MA; ^6^ Program in Medical and Population Genetics Broad Institute of MIT and Harvard Cambridge MA; ^7^ Department of Medical Genetics School of Clinical Medicine University of Cambridge UK; ^8^ The Alan Turing Institute London UK; ^9^ Medical Research Council Biostatistics Unit University of Cambridge UK; ^10^ Stroke Pharmacogenomics and Genetics Group Biomedical Research Institute Sant Pau Spain; ^11^ Department of Public Health and Primary Care Cardiovascular Epidemiology Unit University of Cambridge UK; ^12^ Department of Neurology Brigham and Women’s Hospital Boston MA; ^13^ Clinical Pharmacology and Therapeutics Section Institute of Medical and Biomedical Education and Institute for Infection and Immunity St George’s University of London London UK; ^14^ Clinical Pharmacology Group, Pharmacy and Medicines Directorate St George’s University Hospitals NHS Foundation Trust London UK; ^15^ Novo Nordisk Research Centre Oxford Oxford UK

**Keywords:** COVID‐19, cross‐trait linkage disequilibrium score regression, ischemic stroke, Mendelian randomization, Ischemic Stroke, Genetics, Epidemiology

## Abstract

**Background:**

The relationship between COVID‐19 and ischemic stroke is poorly understood due to potential unmeasured confounding and reverse causation. We aimed to leverage genetic data to triangulate reported associations.

**Methods and Results:**

Analyses primarily focused on critical COVID‐19, defined as hospitalization with COVID‐19 requiring respiratory support or resulting in death. Cross‐trait linkage disequilibrium score regression was used to estimate genetic correlations of critical COVID‐19 with ischemic stroke, other related cardiovascular outcomes, and risk factors common to both COVID‐19 and cardiovascular disease (body mass index, smoking and chronic inflammation, estimated using C‐reactive protein). Mendelian randomization analysis was performed to investigate whether liability to critical COVID‐19 was associated with increased risk of any cardiovascular outcome for which genetic correlation was identified. There was evidence of genetic correlation between critical COVID‐19 and ischemic stroke (r_g_=0.29, false discovery rate [FDR]=0.012), body mass index (r_g_=0.21, FDR=0.00002), and C‐reactive protein (r_g_=0.20, FDR=0.00035), but no other trait investigated. In Mendelian randomization, liability to critical COVID‐19 was associated with increased risk of ischemic stroke (odds ratio [OR] per logOR increase in genetically predicted critical COVID‐19 liability 1.03, 95% CI 1.00–1.06, *P*‐value=0.03). Similar estimates were obtained for ischemic stroke subtypes. Consistent estimates were also obtained when performing statistical sensitivity analyses more robust to the inclusion of pleiotropic variants, including multivariable Mendelian randomization analyses adjusting for potential genetic confounding through body mass index, smoking, and chronic inflammation. There was no evidence to suggest that genetic liability to ischemic stroke increased the risk of critical COVID‐19.

**Conclusions:**

These data support that liability to critical COVID‐19 is associated with an increased risk of ischemic stroke. The host response predisposing to severe COVID‐19 is likely to increase the risk of ischemic stroke, independent of other potentially mitigating risk factors.

**Nonstandard Abbreviations and Acronyms**
Fdr: false discovery rate
IVW: inverse‐variance weighted
LDSC: linkage disequilibrium score regression
MR: Mendelian randomization

**Clinical Perspective**

**What Is New?**

- This study identified evidence of genetic correlation between critical COVID‐19 and ischemic stroke, body mass index and C‐reactive protein.
- In Mendelian randomization, liability to critical COVID‐19 was associated with increased risk of any ischemic stroke, with similar estimates obtained for ischemic stroke subtypes.
- There was no evidence to suggest that genetic liability to ischemic stroke increased the risk of critical COVID‐19.

**What Are the Clinical Implications?**

- These data support that liability to critical COVID‐19 is associated with an increased risk of ischemic stroke.
- The host response predisposing to severe COVID‐19 is likely to increase the risk of ischemic stroke, independent of other potentially mitigating risk factors.

SARS‐CoV‐2 infection is the cause of the COVID‐19 pandemic that has resulted in a health crisis of unprecedented magnitude.^1^, ^2^ While much of the disease burden relates to respiratory failure and sepsis, some studies suggest an increased risk of ischemic stroke.^3^, ^4^, ^5^, ^6^ This has been estimated to be seven times greater than in influenza infection,^3^ with up to 5% of people with severe COVID‐19 suffering stroke.^5^ Strokes that occur in individuals with COVID‐19 are more severe, have poorer outcomes, and higher mortality rates than in those without COVID‐19, despite similar acute management.^6^, ^7^ Indeed, almost two‐fifths of people with COVID‐19 who develop stroke consequently die.^8^ However, some studies do not support an increased risk of stroke in individuals with COVID‐19.^9^, ^10^ Obtaining unbiased estimates for the risk of stroke in people with COVID‐19 is challenging due to difficulty diagnosing mild COVID‐19 and an overall reduction in the rate of admission to hospital with stroke, and minor stroke in particular, during the pandemic,^9^, ^11^ Furthermore, observational studies investigating the association between COVID‐19 and stroke are vulnerable to potential confounding and reverse causation.^3^, ^4^, ^5^, ^6^ For example, there are common risk factors for severe COVID‐19 and stroke, such as obesity and smoking.^12^ Similarly, patients with acute stroke have a dampened immune response and may be more susceptible to severe COVID‐19.^13^

Leverage of genetic data can help overcome some of these issues. Cross‐trait linkage disequilibrium score regression (LDSC) can be used to estimate the genetic correlation between traits. Mendelian randomization (MR) can be employed to investigate whether genetic variants predicting an exposure (such as COVID‐19) also associate with risk of an outcome (such as ischemic stroke).^14^ There are numerous plausible mechanisms by which COVID‐19 may be increasing ischemic stroke risk. COVID‐19 can trigger a cytokine storm with upregulation of pro‐inflammatory signaling and endothelial dysfunction that predisposes to a hypercoagulable state and can lead to thromboembolic events.^15^ Indeed, COVID‐19 also appears to promote the development of other cardiovascular disorders including myocardial injury, myocardial ischemia, arrhythmias, heart failure, and venous thromboembolism.^15^ Furthermore, pre‐existing cardiovascular disease (CVD) is associated with high mortality in people with COVID‐19, which has raised the possibility of a bidirectional interaction between COVID‐19 and the cardiovascular system.^15^ MR analyses also can allow the exploration of such bidirectional relationships.

Elucidating the relationship between COVID‐19 and risk of ischemic stroke could prove important for optimizing prevention and treatment strategies. With this in mind, we performed cross‐trait LDSC to investigate whether there is a genetic correlation between COVID‐19 and ischemic stroke, and followed this up with MR analyses to investigate whether any such statistically significant correlation might be explained by liability to COVID‐19 being associated with increased risk of ischemic stroke.

## Methods

All genetic association data used in this work are publicly accessible. Appropriate patient consent and ethical approval had been obtained in the original studies from which they were obtained (Table S1). Statistical code related to the analyses performed in the current study is freely available from Github (https://github.com/verena‐zuber/covid19_and_stroke).

### Study Overview

First, we performed cross‐trait LDSC to estimate genetic correlations for COVID‐19 with ischemic stroke, other related CVDs, and risk factors common to both COVID‐19 and CVD. Second, for CVD outcomes that showed evidence of genetic correlation with COVID‐19, MR analysis was performed to investigate whether liability to COVID‐19 was also associated with these outcomes. Finally, bidirectional MR was carried out to investigate potential reverse associations, ie whether genetic liability to the CVD outcome was also associated with increased risk of COVID‐19. A graphical overview of the analysis plan is presented in Figure S1.

### Exposure Definitions and Genetic Association Estimates for COVID‐19

Genetic association estimates for COVID‐19 were obtained from release 5 of the COVID‐19 host genetics consortium.^16^, ^17^ In our main analysis we focused on the most severe definition of COVID‐19 available (referred to *critical COVID‐19* from here), where a critical case is defined as an individual who was hospitalized with laboratory confirmed SARS‐CoV‐2 infection and required respiratory support or died. Genetic associations were derived from 5101 cases and 1 383 241 controls from the general population. Hospital admission and requiring respiratory support or death is a proxy for disease severity and is preferred here over other case definitions which are solely based on a positive COVID‐19 test result. Previous studies have shown that bias may impact analyses identifying cases based on likelihood of testing for SARS‐CoV‐2 infection, because participants being tested for SARS‐CoV‐2 infection are selected for a wide range of genetic, behavioral, and demographic traits.^9^

Results based on other COVID‐19 definitions from the COVID‐19 host genetics consortium were performed as further sensitivity analysis. As the first sensitivity analysis definition, we compared individuals with laboratory confirmed SARS‐CoV‐2 infection who had been hospitalized (cases) versus individuals with laboratory confirmed SARS‐CoV‐2 infection who did not require hospitalization (4829 cases and 11 816 controls). As a second analysis sensitivity definition, we compared individuals with laboratory confirmed SARS‐CoV‐2 infection who had been hospitalized (cases) versus the general population (9986 cases and 1 877 672 controls). The third sensitivity analysis definition was based on individuals with reported COVID‐19 (laboratory confirmed, physician‐reported or self‐reported; cases) versus controls from the general population (38 984 cases and 1 644 784 controls). An overview of the COVID‐19 definitions is given in Table S1.

### Outcomes

#### Ischemic Stroke

The primary outcome was any ischemic stroke (34 217 cases). In secondary hypothesis‐generating analyses, stroke subtypes were further explored as large artery stroke (LAS, 4373 cases), cardioembolic stroke (CES, 7193 cases), and small vessel stroke (SVS, 5386 cases).^18^ The common control pool included 406 111 individuals. Genetic association data were derived from the MEGASTROKE consortium.^18^

#### Related CVD Outcomes

We considered other CVD outcomes related to ischemic stroke in their pathophysiology. These were coronary artery disease (including myocardial infarction, acute coronary syndrome, chronic stable angina, or >50% coronary artery stenosis), heart failure, and atrial fibrillation. Genetic associations with risk for coronary artery disease were measured on 60 801 cases and 123 504 controls and taken from the Coronary ARtery DIsease Genome wide Replication and Meta‐analysis (CARDIOGRAM) plus The Coronary Artery Disease (C4D) consortium (CARDIoGRAMplusC4D),^19^ for heart failure were measured on 47 309 cases and 930 014 controls and taken from the HEart failuRe Molecular Epidemiology for therapeutic targetS (HERMES) consortium^20^ and for atrial fibrillation were measured on 65 446 cases and 522 744 controls and taken from a transethnic meta‐analysis.^21^

### Risk Factors Related to Both COVID‐19 and CVD

To investigate whether any genetic correlation between critical COVID‐19 and the CVD outcomes was related to confounding factors, we further considered common risk factors to both, including obesity, smoking, and chronic inflammation.^22^, ^23^, ^24^, ^25^ Genetic association estimates to proxy these traits were taken from a genome‐wide association study (GWAS) on body mass index (BMI) measured on 694 649 subjects,^26^ lifetime smoking index measured on 462 690 subjects,^27^ and CRP (C‐reactive protein) measured on 361 194 individuals in UK Biobank.^28^

### Statistical Analysis

#### Cross‐Trait Linkage Disequilibrium Score Regression

We performed LDSC to estimate the genetic correlation (r_g_) of critical COVID‐19 with the primary outcome ischemic stroke, and secondary outcomes coronary artery disease, heart failure, and atrial fibrillation, using GWAS summary statistics data.^29^ We also estimated correlation with possible genetic confounders, including BMI, lifetime smoking index, and CRP. We restricted our analyses to HapMap 3 single‐nucleotide polymorphisms (SNPs), which are known to be well‐imputed across most studies and utilized the pre‐computed European LD‐scores estimated using the 1000G reference panel, provided by the LDSC creators. For each set of summary statistics, the SNP‐specific sample size information was used. If not available, we assumed that all SNPs had the same sample size for that trait, defined as the total sample size for continuous phenotypes or as the sum of cases and controls for case/control phenotypes. By default, LDSC also removed variants that were duplicate, strand‐ambiguous, not SNPs (eg indels), with *P*‐values not between 0 and 1, with alleles that did not match with the 1000G reference panel, and with low effective sample size or not included in all studies of a GWAS meta‐analysis (if such information was available) for traits with no effective sample size information. After estimation of the genetic correlation across all phenotypes, we corrected for multiple hypothesis testing using the Benjamini and Yekutieli false discovery rate (FDR).^30^ FDR‐corrected *P*‐values <0.05 were considered statistically significant.

#### Mendelian Randomization Analyses

##### Genetic Variants Used as Instrumental Variables

Genetic variants were selected based on associations with critical COVID‐19. In our main analysis, we selected uncorrelated genetic variants (clumped at correlation threshold *r^2^<*0.01) at *P*‐value <5×10^−6^. In sensitivity analyses, we applied a more stringent threshold and considered only genome‐wide significant genetic variants (*P*‐value <5×10^−8^).

##### Main Analysis

For CVD outcomes that showed evidence of genetic correlation with critical COVID‐19 in LDSC, MR analysis was performed to estimate the association of genetically predicted liability to critical COVID‐19 with that outcome using the random effects 2‐sample inverse‐variance weighted (IVW) method.^31^ The IVW estimate can be biased by pleiotropy when a genetic variant associates the outcome (eg, ischemic stroke) via a pathway other than through the exposure (ie, liability to critical COVID‐19). Pleiotropy can cause heterogeneity in the MR estimates obtained by different variants employed as instruments, which was assessed using the *Q‐*statistic and the respective heterogeneity *P‐*value.^32^ A Mendelian randomization estimate with *P‐*value <0.05 for the main IVW analysis was deemed to represent supportive evidence, given that MR was only performed to follow up positive LDSC findings.

##### Sensitivity Analyses—Robust Methods

We performed sensitivity analyses with pleiotropy‐robust two‐sample summary‐level MR approaches, including the weighted median MR^33^ and MR‐Egger^34^ to compare the MR estimates between different MR models. Each of these methods provides a statistically consistent estimator of the true causal estimate under different assumptions. The intercept of the MR‐Egger model represents a test for directional pleiotropy and we included this in sensitivity analyses.^35^

##### Pleiotropic Pathways—Inflammation and Cardiometabolic Risk Factors

We further performed multivariable MR to adjust for potential pleiotropic pathways via cardiometabolic risk factors that are known to affect risk of both COVID‐19 and CVD12, including obesity (BMI),^26^ lifetime smoking index,^27^ and chronic inflammation (estimated using CRP). Multivariable MR includes all the respective genetic associations in a joint model to account for genetic confounding.^36^ While univariable MR measures the total estimate of an exposure, multivariable MR measures the direct estimate of the exposure independent of other risk factors (ie, pleiotropy or genetic confounders) in the model.^37^ In the multivariable MR model, we selected instruments based on the primary exposure of critical COVID‐19. We compared the multivariable MR model with the univariable MR model using likelihood ratio test to evaluate if accounting for the pleiotropic pathway provides a better model fit than the univariable MR model.

##### Bidirectional MR

For CVD outcomes that showed evidence of genetic correlation with critical COVID‐19 in LDSC, bidirectional MR was also performed to investigate for any association of genetic liability to that CVD outcome with risk of critical COVID‐19. Uncorrelated genetic variants (*r^2^<*0.01) associated with the CVD outcome at a *P*‐value <5×10^−6^ were selected as instruments.

##### Power Calculation

Power to detect an association of genetically proxied liability to critical COVID‐19 with all‐cause ischemic stroke and its subtypes was assessed using power calculations for MR.^38^ Sample sizes were set according to the number of cases and controls in the MEGASTROKE consortium as given in Table S1. Heritability of the exposure was calculated from the *F‐*statistic of the genetic variants selected as instrumental variables, which was approximated using the squared regression coefficient divided by its squared standard error. The *F‐*statistic was then transformed to the proportion of variance in the phenotype explained by the genetic variants *R^2^* using the Cragg–Donald transformation.^39^

MR estimates are expressed as odds ratios (OR) per unit increase in the logOR of the exposure for binary traits. All analyses were performed using the ieugwasr (version 0.1.5) and MendelianRandomization (version 0.5.0) R packages.^40^

## Results

### LD Score Regression

**Figure 1:**
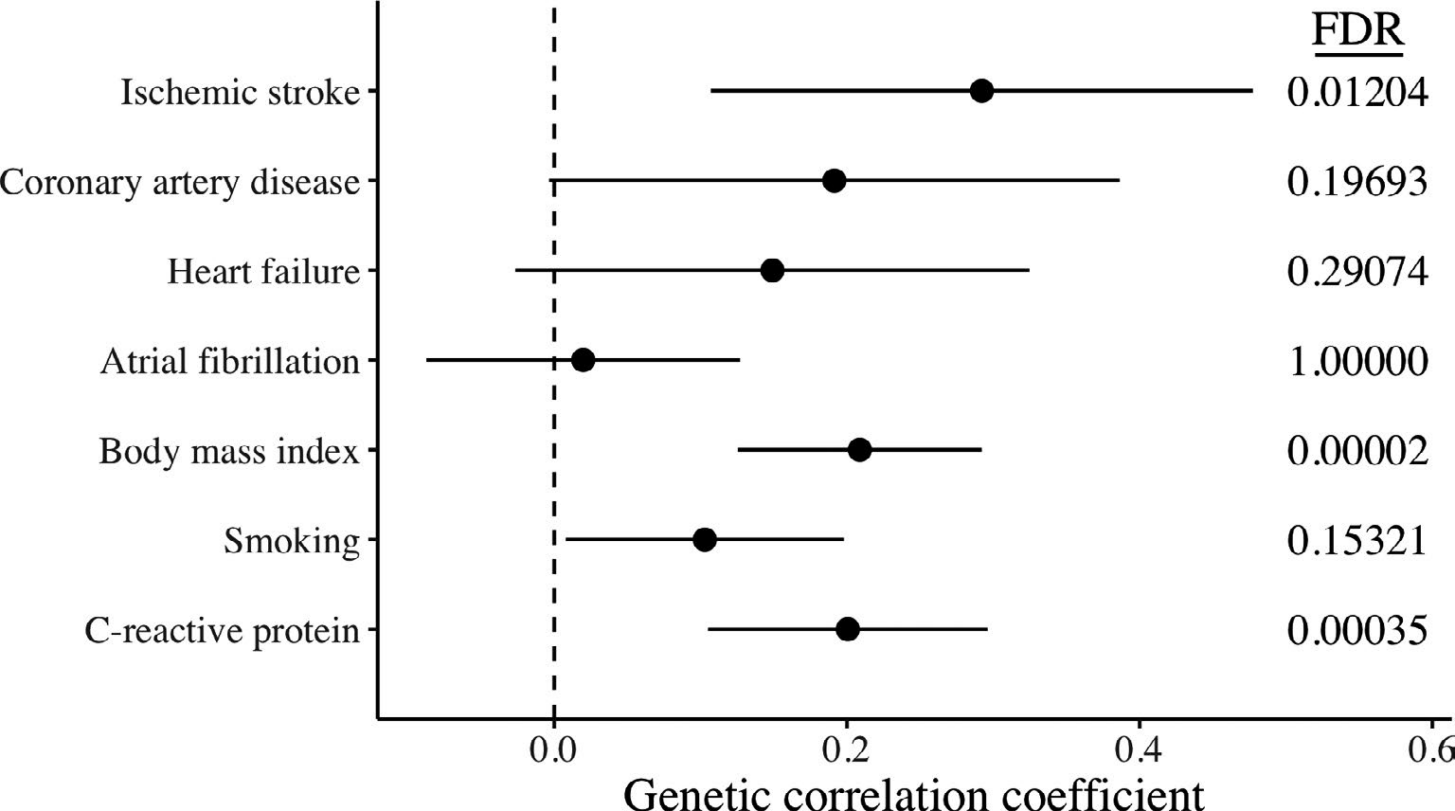
Genetic correlation coefficient (*x*‐axis) with 95% CI for critical COVID‐19 and ischemic stroke, related cardiovascular disease outcomes, and risk factors for both COVID‐19 and cardiovascular disease (*y*‐axis), estimated by cross‐trait linkage disequilibrium score regression. Multiple testing adjustment using the Benjamini and Yekutieli false discovery rate (FDR) are given on the right.

Performing LDSC, we found evidence of genetic correlation between critical COVID‐19 and ischemic stroke (r_g_=0.29, FDR‐*P*‐value=0.012) (Figure 1). Critical COVID‐19 was also genetically correlated with BMI (r_g_=0.21, FDR‐*P*‐value=0.00002) and CRP (r_g_=0.20, FDR‐*P*‐value=0.00035). We did not observe evidence for genetic correlation between critical COVID‐19 and the CVD outcomes (Table S2), and therefore focused the consequent MR analysis only on ischemic stroke and its subtypes.

### Mendelian Randomization

**Figure 2:**
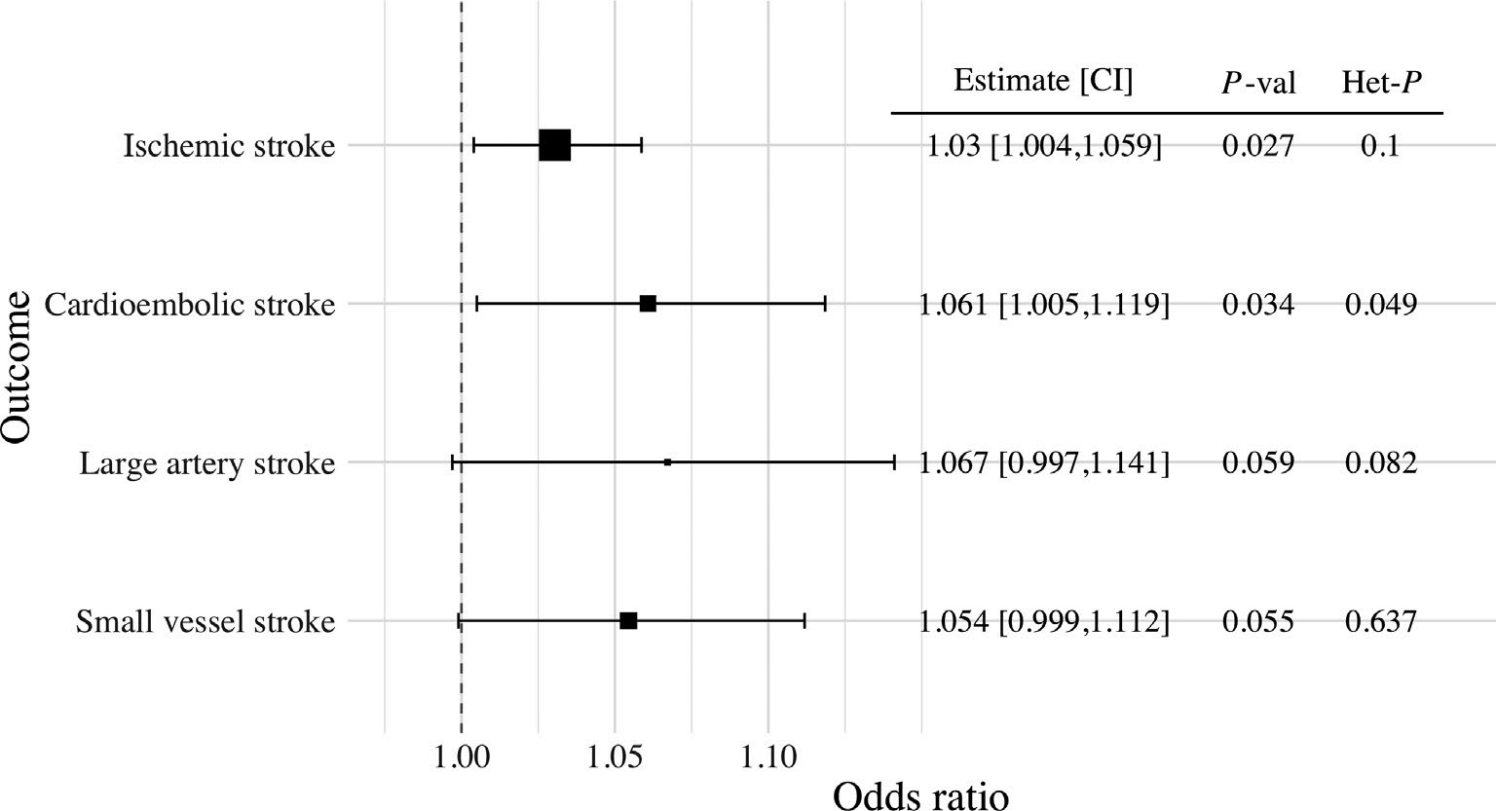
Forest plot illustrating the Mendelian randomization estimates of liability to critical COVID‐19 with stroke outcomes based on inverse‐variance weighted Mendelian randomization using genetic variants that were associated with critical COVID‐19 at a *P*‐value level of 5×10^‐6^ or smaller. Mendelian randomization estimates represent the odds ratio of ischemic stroke outcomes per unit increase in the log‐odds ratio of liability to critical COVID‐19. Additional columns include the Mendelian randomization estimate, its 95% CI, the *P*‐value of the inverse‐variance weighted Mendelian randomization estimate to be different from 1 (*P*‐val), and heterogeneity measured by the Q‐statistic and the respective heterogeneity *P*‐value (Het‐*P*). Outcomes included any ischemic stroke, cardioembolic stroke, large artery stroke, and small vessel stroke.

We selected 31 uncorrelated genetic variants as instrumental variables for liability to critical COVID‐19. These are detailed in Table S3, along with their associations with ischemic stroke and its subtypes. MR estimates are presented in Figure 2. In a univariable MR analysis, genetically proxied liability to critical COVID‐19 was associated with all‐cause ischemic stroke (OR 1.03, 95% CI 1.00 to 1.06, *P*‐value=0.03). Restricting to ischemic stroke subtypes, there were similar MR estimates for cardioembolic stroke (OR 1.06, 95% CI 1.01 to 1.12, *P*‐value=0.03), large artery stroke (OR 1.07, 95% CI 1.00 to 1.14, *P*‐value=0.06) and small‐vessel stroke (OR 1.05, 95% CI 1.00 to 1.11, *P*‐value=0.06). Power calculations shown in Figure S2 supported lower power for ischemic stroke subtypes compared to all‐cause ischemic stroke. For the MR estimates generated by different variants, we observed heterogeneity greater than would be expected by chance only for cardioembolic stroke (heterogeneity *P*‐value=0.049), but none of the other considered ischemic stroke categories.

### Mendelian Randomization Sensitivity Analyses

Diagnostic scatterplots for ischemic stroke outcomes are presented in Figure S3. We observed consistent MR estimates for ischemic stroke risk in sensitivity analyses based on pleiotropy‐robust approaches as in the main analysis and none of the intercept estimates of MR‐Egger suggested directional pleiotropy (Table S4). In multivariable MR to investigate potential pleiotropy through risk factors common to both COVID‐19 and CVD, there was little evidence for attenuation of the size of the estimate in any of these analyses (Figure S4), which was confirmed by likelihood ratio test (Table S5).

### Sensitivity Analysis Based on Genome‐Wide Significant Genetic Variants

As an additional sensitivity analysis, we used a more stringent *P‐*value threshold based on genome‐wide significance to select genetic variants as instrumental variables. We identified 9 uncorrelated genetic variants that associated with critical COVID‐19 at genome‐wide significance (*P‐*value <5×10^−8^ *)*. This MR analysis based on fewer variants generated consistent estimates to the main analysis, but with wider CIs that crossed the null, reflective of lower statistical power. Results are displayed in Figure S5.

### Comparison With Other COVID‐19 Definitions

We further considered other COVID‐19 definitions (Figure S6 and Table S6). Genetically predicted COVID‐19 requiring hospitalization as compared to not requiring hospitalization was associated with increased risk of any ischemic stroke (OR 1.05, 95% CI 1.01 to 1.10, *P*‐value=0.01) and small‐vessel stroke (OR 1.22, 95% CI 1.11 to 1.34, *P*‐value=0.000055). Considering reported COVID‐19 (laboratory confirmed, physician‐reported or self‐reported) versus controls from the general population, this was associated with increased risk of any ischemic stroke (OR 1.13, 95% CI 1.01 to 1.26, *P*‐value=0.04) and large artery stroke (OR 1.46, 95% CI 1.18 to 1.81, *P*‐value=0.00042).

### Bidirectional MR

**Figure 3:**
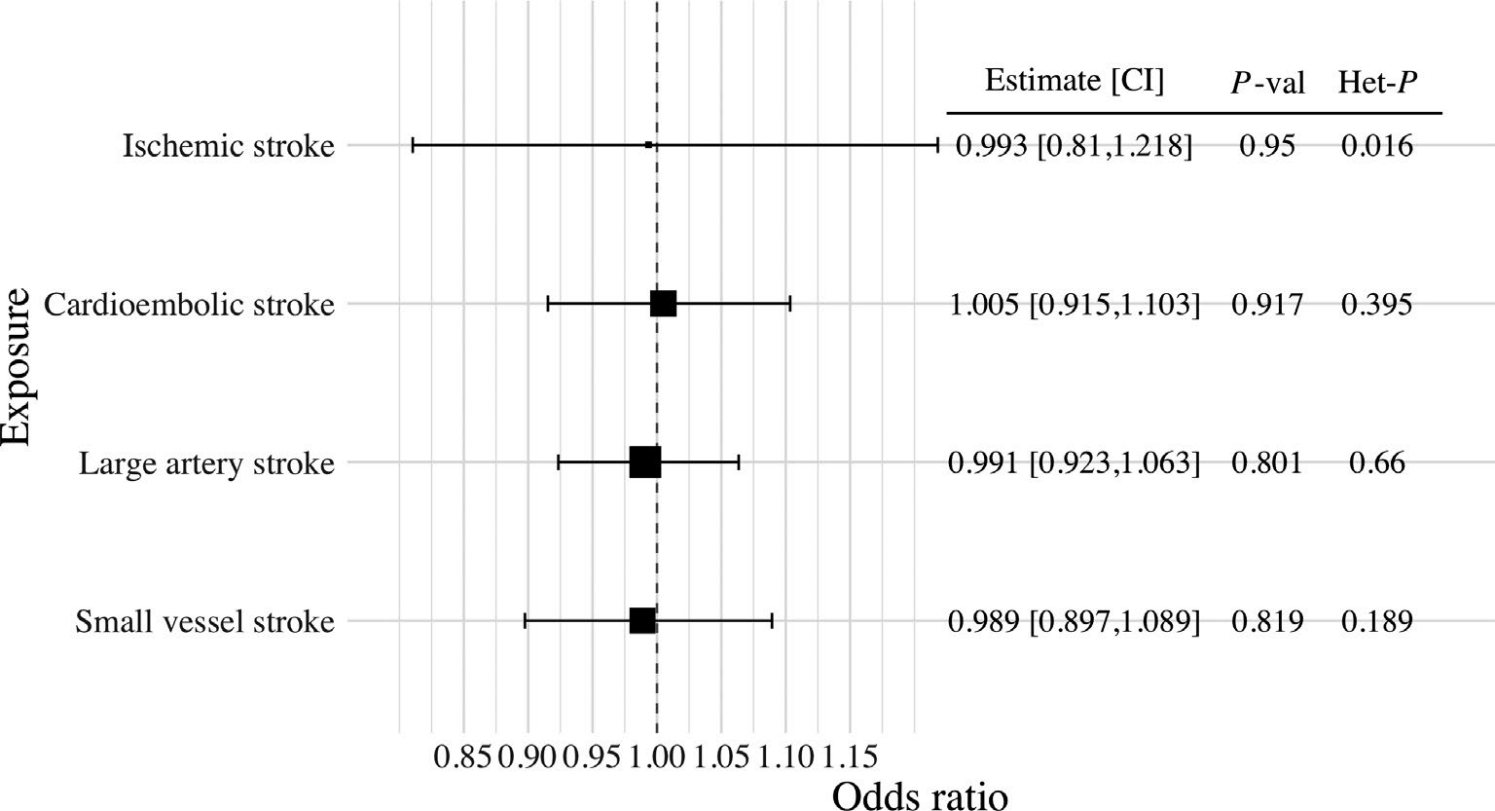
Forest plot of the bidirectional Mendelian randomization analysis illustrating the inverse‐variance weighted Mendelian randomization estimate of liability to stroke phenotypes with critical COVID‐19. Genetic variants which were associated with the stroke phenotypes were selected as instrumental variables at a *P*‐value level of 5×10^‐6^ or smaller. Mendelian randomization estimates represent the odds ratio of critical COVID‐19 per unit increase in the log odds ratio of stroke phenotype. Additional columns include the Mendelian randomization estimate, its 95% CI, the *P*‐value of the inverse‐variance weighted Mendelian randomization estimate to be different from 1 (*P*‐val), and heterogeneity measured by the Q‐statistic and the respective heterogeneity *P*‐value (Het‐*P*). Exposures included any ischemic stroke, cardioembolic stroke, large artery stroke, and small vessel stroke.

There was no strong evidence to support that genetic liability to any of the considered ischemic stroke outcomes was associated with increased risk of critical COVID‐19, as illustrated in Figure 3.

## Discussion

In this study, we used cross‐trait LDSC to explore the genetic correlation of critical COVID‐19 with ischemic stroke, other CVD outcomes, and risk factors common to both. We identified a genetic correlation between critical COVID‐19 and ischemic stroke, and performed MR analyses that found genetic liability to critical COVID‐19 to be associated with increased risk of ischemic stroke. Notably, there was no evidence to support that these associations were attributable to shared risk factors, such as obesity, smoking, and chronic inflammation. Furthermore, there was no MR evidence that genetic liability to ischemic stroke increases risk of critical COVID‐19.

When we considered critical COVID‐19 and ischemic stroke subtypes, only cardioembolic stroke remained statistically significant. There are 2 main possible explanations for this observation. The first is that there are pathophysiological differences across stroke subtypes. Indeed, it is conceivable that the host response to critical COVID‐19 may be more likely to culminate in cardioembolic stroke through an acute, pro‐inflammatory, hypercoagulable state that results in cardiac thromboembolism; as opposed to occlusion of small penetrating cerebral arteries and small vessel strokes which are generally a result of more longstanding conditions such as hypertension or diabetes.^41^ The second possible explanation is that smaller sample sizes for each stroke subtype compared to any ischemic stroke may mean that we were unable to detect a statistically significance difference in stroke subtypes due to reduced power as shown in Figure S2. This explanation would be supported by similar point estimates to any ischemic stroke across stroke subtypes.

To date, studies assessing the incidence of ischemic stroke during the COVID‐19 pandemic have produced contrasting findings. It has also been uncertain whether any association between COVID‐19 and ischemic stroke is due to a direct effect of SARS‐CoV‐2 viral infection, the host response in COVID‐19, or both. On one hand, some studies demonstrate that the likelihood of stroke is 7‐fold higher in people with COVID‐19 than with influenza,^3^ that COVID‐19 is associated with 21‐fold increased odds of in‐hospital stroke compared to patients without COVID‐19,^6^ and that stroke is the most common neurological/neuropsychiatric complication of COVID‐19.^4^ On the contrary, other studies have demonstrated a reduced rate of hospital admissions with stroke during the first wave of the pandemic compared to one year before.^9^ Two main hypotheses have been proposed as explanations for these contrasting findings. The first is that the incidence of stroke declined during the first wave of the pandemic and that COVID‐19 is not mechanistically associated with stroke, and the second is that the observed reduction in stroke presentations was due to a higher proportion of people with mild strokes not reaching stroke services.^11^, ^42^

We have leveraged large‐scale genetic data to address this and find that liability to critical COVID‐19 is associated with increased risk of ischemic stroke. Moreover, our results are consistent with the hypothesis that it is the host response in COVID‐19 which contributes to increased ischemic stroke risk. However, it is also important to note that our study design cannot directly inform on whether the SARS‐CoV‐2 virus itself also be increases ischemic stroke risk, irrespective of the host inflammatory response. Mechanisms that increase risk of ischemic stroke in patients with COVID‐19 are complex,^5^, ^15^ and include systemic inflammation and endotheliopathy.^15^, ^43^, ^44^, ^45^ COVID‐19 can trigger a cytokine storm with upregulation of pro‐inflammatory cytokines and chemokines such as tumor necrosis factor‐α (TNF‐α), interleukin‐1 (IL‐1) and IL‐615. Endothelial inflammation can induce a microvascular and macrovascular endotheliopathy that contributes to a pro‐thrombotic state.^15^, ^43^

While prophylactic low molecular weight heparin is used to prevent thromboembolism in patients with COVID‐19, more targeted approaches to prevent strokes are yet undefined.^5^, ^46^ Moreover, the REMAP‐CAP (A Randomised, Embedded, Multi‐factorial, Adaptive Platform Trial for Community‐Acquired Pneumonia), ACTIV‐4 (Anti‐thrombotics for Adults Hospitalized With COVID‐19), and ATTACC (Antithrombotic Therapy to Ameliorate Complications of COVID‐19) trials have recently reported that therapeutic doses of anticoagulation do not improve clinical outcome and may increase bleeding for people with COVID‐19 in the critical care setting. Previous work using an MR approach anticipated a beneficial effect of IL‐6 receptor inhibition on both risk of ischemic stroke and severe COVID‐19.^47^, ^48^ More recently, clinical trials have demonstrated that IL‐6 receptor inhibition can improve outcomes in patients hospitalized with COVID‐19.^49^ Targeting the deleterious host immune response through similar approaches may also help to reduce the risk of ischemic stroke and should be further evaluated.

Our findings also support the hypothesis that few patients with minor strokes reached stroke services during the first wave of the COVID‐19 pandemic.^11^ This is reinforced by data that demonstrate the reduction in stroke admissions observed in some centers during the first wave of the pandemic was driven mainly by a reduction in presentations with minor stroke syndromes.^11^ People with minor stroke are at high risk of early recurrence^50^ and public health messaging should encourage people to attend stroke services if they have any symptom of stroke during the COVID‐19 pandemic.

We did not observe genetic correlation between critical COVID‐19 and other CVD outcomes such as coronary heart disease, heart failure or atrial fibrillation. Other studies have reported acute coronary syndrome, heart failure, and arrhythmia in people with COVID‐19.^15^ There are a number of possible explanations for these findings, which at first may appear discordant. First, myocardial injury and myocarditis may be a more common cardiac manifestation of COVID‐19 than coronary artery plaque rupture and thrombosis. Second, the pulmonary oedema that is observed in people with critical COVID‐19 is usually accompanied by acute respiratory distress syndrome and is mainly regarded as non‐cardiogenic.^15^ Third, while arrhythmias are common manifestations of COVID‐19, these may be triggered by acute myocardial injury and systemic factors (such as fever, sepsis, hypoxia, and electrolyte imbalance), rather than atrial fibrillation due to a chronic atrial cardiopathy, which is more likely to make up cases in the pre‐pandemic atrial fibrillation GWAS.^20^

Our current study has strengths. We have made efficient use of existing large‐scale data resources to address an important clinical issue in the context of the rapidly evolving global pandemic. A key strength of MR analysis is the use of randomly allocated genetic variants to help overcome environmental confounding, which is analogous to randomization of treatment allocation in clinical trials. This has helped to overcome some of the limitations of previous observational studies (either retrospective or cross‐sectional) assessing the relationship between COVID‐19 and ischemic stroke.^3^, ^4^, ^5^, ^6^

Our work also has limitations. A series of modelling assumptions are made when using MR, in particular, that the genetic variants do not affect the considered outcomes through pathways independent of the exposure. While this can never be completely excluded, we employed methods that are robust to genetic confounding (pleiotropy) in a series of sensitivity analyses (including pleiotropy‐robust MR methods and accounting for measured pleiotropy using multivariable MR) and the estimates were consistent with our main analyses. We cannot be certain that genetic associations with liability to critical COVID‐19 accurately reflect the pathophysiological process that actually occurs during critical COVID‐19. For example, while genetic predisposition may place an individual at increased liability to critical COVID‐19, it is not possible to determine from our analyses whether that factor is involved in the pathophysiological response to COVID‐19.

In conclusion, we have found genetic evidence that liability to critical COVID‐19 is associated with increased risk of ischemic stroke. Our results are consistent with the host response in critical COVID‐19 underlying this relationship, and support the evaluation of strategies to mitigate this.

## Sources of Funding

VZ is supported by UK Dementia Research Institute at Imperial College London, which is funded by the Medical Research Council, Alzheimer’s Society and Alzheimer’s Research UK (MC_PC_17114). The research leading to these results has been conducted as part of the COVIRNA project (grant agreement n°101016072) funded by the European Union’s Horizon 2020 Framework Programme for Research and Innovation. EPM is supported by the National Institutes of Health of the United States (R01NS103924, U01NS069763). LB acknowledges the MRC grant MR/S02638X/1, The BHF‐Turing Cardiovascular Data Science Awards 2017 and The Alan Turing Institute under the Engineering and Physical Sciences Research Council grant EP/N510129/1. IF‐C is supported by Inmungen‐Cov2 project, Centro Superior de Investigaciones Científicas (CSIC). SB is supported by a Sir Henry Dale Fellowship jointly funded by the Wellcome Trust and the Royal Society (204623/Z/16/Z). This research was supported by the UKRI Medical Research Council [MC_UU_00002/7] and the NIHR Cambridge Biomedical Research Centre (BRC‐1215‐20014). The views expressed are those of the author(s) and not necessarily those of the NIHR or the Department of Health and Social Care. CDA is supported by the National Institutes of Health of the United States (R01NS103924, U01NS069763). DG is supported by the British Heart Foundation Centre of Research Excellence at Imperial College London (RE/18/4/34215) and by a National Institute for Health Research Clinical Lectureship at St. George's, University of London (CL‐2020‐16‐001). This research was funded in part by the Wellcome Trust.

## Disclosures

CDA receives sponsored research support from the American Heart Association, Massachusetts General Hospital, and Bayer AG, and has consulted for ApoPharma, Inc. DG is employed part‐time by Novo Nordisk. The remaining authors have no disclosures to report.

## Supporting information

Table S1–S6
Figure S1–S6
