# Supplementary material for: Leveraging Genetic Data to Elucidate the Relationship Between COVID‐19 and Ischemic Stroke
Source: J Am Heart Assoc. 2021 Nov 10;10(22):e022433. doi: 10.1161/JAHA.121.022433 (PMC8751930; doi:10.1161/JAHA.121.022433)
Supplement: Supplementary file 1 — Table S1–S6 Figure S1–S6 [file JAH3-10-e022433-s001.pdf]

Supplemental Material

Table S1: Overview of the publicly available summary-level data of genetic associations used for the analysis.

Phenotype	Description	Sample Size	Cases	Controls	Population	Pubmed	Author
**Covid-19**	Main: Critical Covid *vs.* population		5,101	1,383,241	EUR		The COVID-19 Host Genetics Initiative (2021)
	Sensitivity Analysis 1: Hospitalized Covid *vs.* not hospitalized Covid		4,829	11,816			
	Sensitivity Analysis 2: Hospitalized Covid *vs.* population		9,986	1,877,672			
	Sensitivity Analysis 3: Covid vs population		38,984	1,644,784			
**Stroke**	Any ischemic stroke		34,217	406,111	EUR	29531354	Malik et al. (2018)
	Large artery stroke		4,373	406,111			
	Cardioembolic stroke		7,193	406,111			
	Small vessel stroke		5,386	406,111			
**Cardiovascular disease outcomes**	Coronary artery disease		60,801	123,504	EUR, SEA	26343387	Nikpay et al. (2015)
	Heart failure		47,309	930,014	EUR	31919418	Shah et al. (2020)
	Atrial fibrillation		65,446	522,744	TRANS	29892015	Roselli et al. (2018)
**Obesity**	Body mass index	694,649			EUR	30239722	Pullit et al. (2019)
**Smoking**	Lifetime smoking index	462,690			EUR	30239722	Wootton et al. (2020)
**Inflammation**	C-reactive protein	361,194			EUR		Neale lab

Table S2. Cross-trait linkage disequilibrium score regression (LDSC) analysis results of critical Covid-19 with ischemic stroke, cardiovascular disease (CVD) outcomes, and risk factors related to both Covid-19 and CVD. r_g represents the genetic correlation between critical Covid-19 and each phenotype. *p*-values are corrected for multiple testing with the Benjamini and Hochberg false discovery rate (FDR).

	r_g	FDR-*p* value
**Ischemic stroke**	0.2922	4.65E-03
**Coronary artery disease**	0.1914	7.60E-02
**Heart failure**	0.1491	1.12E-01
**Atrial fibrillation**	0.0198	7.17E-01
**Body mass index**	0.2088	6.26E-06
**Smoking**	0.1029	5.91E-02
**C-reactive protein**	0.2006	1.35E-04

Table S3: Overview of the genetic variants used as instrumental variables for liability to critical Covid-19 based on the Covid-19 host genetics initiative. We selected 31 uncorrelated (clumped at correlation threshold $r^2 < 0.01$) genetic variants as instrumental variables for laibility to critical Covid-19 that were associated at a *p*-value level of 5×10^{-6} or smaller. The table additionally includes summary-level data (beta coefficients of genetic association, their standard error and corresponding *p*-value) for liability to critical Covid-19 as exposure and any ischemic stroke (AIS) and its subtypes cardioembolic stroke (CES), large artery stroke (LAS), and small vessel stroke (SVS) as outcomes. Alt: alternative allele; Chr: chromosome; Pos: position; Ref: reference allele; SE: standard error.

					**Critical Covid-19**			**AIS**			**CES**			**LAS**			**SVS**		
**Variant**	**Chr**	**Pos**	**Ref**	**Alt**	**Beta**	**SE**	**P**	**Beta**	**SE**	**P**	**Beta**	**SE**	**P**	**Beta**	**SE**	**P**	**Beta**	**SE**	**P**
rs10087754	8	121819908	T	A	-1.3E-01	2.7E-02	7.1E-07	-1.2E-02	1.0E-02	2.4E-01	1.0E-04	1.9E-02	9.9E-01	-4.2E-02	2.5E-02	9.6E-02	-9.6E-03	2.3E-02	6.8E-01
rs11085727	19	10355447	C	T	1.7E-01	2.9E-02	3.7E-09	-2.2E-02	1.1E-02	5.2E-02	-5.5E-02	2.2E-02	1.1E-02	-5.0E-04	2.8E-02	9.8E-01	3.5E-02	2.5E-02	1.7E-01
rs111508230	1	155181061	C	T	-2.1E-01	4.4E-02	2.6E-06	-8.1E-03	1.6E-02	6.1E-01	-7.1E-02	3.1E-02	2.4E-02	9.3E-02	3.9E-02	1.7E-02	2.9E-02	4.1E-02	4.8E-01
rs114969787	5	65770656	C	T	3.1E-01	6.6E-02	3.6E-06	4.1E-02	3.1E-02	1.9E-01	-4.7E-02	6.4E-02	4.7E-01	1.2E-01	7.7E-02	1.4E-01	4.4E-02	7.2E-02	5.5E-01
rs11658357	17	36097317	A	T	-2.0E-01	4.4E-02	4.9E-06	9.7E-03	1.3E-02	4.4E-01	-1.8E-02	2.4E-02	4.5E-01	-1.5E-02	3.1E-02	6.1E-01	4.4E-02	2.9E-02	1.3E-01
rs117232645	13	74553195	G	A	-3.3E-01	7.0E-02	2.5E-06	-3.1E-02	3.0E-02	3.0E-01	1.0E-02	5.9E-02	8.6E-01	4.4E-02	7.3E-02	5.4E-01	3.8E-03	6.8E-02	9.6E-01
rs13050728	21	33242905	T	C	-2.0E-01	2.9E-02	2.4E-12	-4.4E-03	1.1E-02	6.8E-01	-2.9E-02	2.0E-02	1.5E-01	-2.7E-02	2.6E-02	3.1E-01	2.7E-02	2.5E-02	2.8E-01
rs13080258	3	69672908	A	C	-1.4E-01	3.0E-02	3.6E-06	2.8E-03	1.2E-02	8.1E-01	6.8E-03	2.3E-02	7.6E-01	-1.6E-02	2.9E-02	5.9E-01	-1.8E-02	2.7E-02	5.1E-01
rs13274496	8	22583385	G	A	-2.0E-01	4.2E-02	1.8E-06	7.0E-04	1.3E-02	9.6E-01	1.7E-02	2.4E-02	4.8E-01	-1.9E-02	3.2E-02	5.5E-01	-3.2E-02	2.9E-02	2.6E-01
rs143334143	6	31153649	G	A	2.9E-01	4.3E-02	6.0E-12	9.0E-03	1.9E-02	6.4E-01	3.6E-02	3.6E-02	3.1E-01	-1.0E-02	4.8E-02	8.3E-01	3.6E-02	4.6E-02	4.3E-01
rs1974792	19	50353078	A	G	-1.4E-01	2.7E-02	5.1E-07	-2.0E-02	1.0E-02	4.7E-02	-9.6E-03	2.0E-02	6.3E-01	-6.3E-02	2.5E-02	1.3E-02	3.2E-02	2.4E-02	1.8E-01
rs2109069	19	4719431	G	A	2.6E-01	2.8E-02	6.1E-20	-7.1E-03	1.1E-02	5.2E-01	2.0E-03	2.2E-02	9.3E-01	-6.2E-03	2.7E-02	8.2E-01	-1.1E-02	2.6E-02	6.8E-01
rs2237698	7	107967457	C	T	2.4E-01	4.0E-02	2.4E-09	-2.7E-02	2.1E-02	1.9E-01	-2.7E-02	3.9E-02	4.8E-01	4.8E-02	5.1E-02	3.5E-01	-2.3E-02	4.7E-02	6.3E-01
rs2597569	11	97922951	T	C	-1.8E-01	3.4E-02	9.3E-08	-7.0E-03	1.0E-02	4.9E-01	-1.5E-02	1.9E-02	4.5E-01	-3.3E-02	2.5E-02	2.0E-01	-3.5E-02	2.4E-02	1.4E-01
rs2733839	12	10393411	T	C	2.7E-01	5.8E-02	3.5E-06	-3.9E-03	2.7E-02	8.9E-01	4.3E-02	5.3E-02	4.2E-01	-4.1E-02	7.2E-02	5.7E-01	3.0E-02	6.4E-02	6.4E-01
rs340850	1	213941523	T	G	-2.8E-01	6.0E-02	4.3E-06	2.0E-02	2.4E-02	4.0E-01	5.6E-02	4.8E-02	2.5E-01	-9.7E-02	5.7E-02	9.0E-02	-5.3E-02	5.5E-02	3.3E-01
rs35081325	3	45848429	A	T	6.3E-01	4.5E-02	5.8E-45	3.0E-02	2.0E-02	1.3E-01	4.3E-02	3.8E-02	2.6E-01	5.4E-02	5.1E-02	2.8E-01	2.7E-02	4.9E-02	5.9E-01
rs36932	7	123877938	G	A	-1.6E-01	3.4E-02	4.0E-06	-6.2E-03	1.4E-02	6.5E-01	-2.1E-02	2.6E-02	4.3E-01	-3.2E-02	3.3E-02	3.3E-01	-4.8E-02	3.1E-02	1.2E-01
rs4076440	1	9630418	A	G	2.1E-01	4.3E-02	9.4E-07	-1.5E-02	2.1E-02	4.7E-01	4.2E-03	4.1E-02	9.2E-01	-5.8E-02	5.3E-02	2.7E-01	5.1E-03	4.8E-02	9.2E-01
rs5767981	22	47769327	A	G	-1.6E-01	3.4E-02	5.0E-06	-2.0E-02	1.1E-02	5.7E-02	-7.9E-03	2.1E-02	7.0E-01	-3.0E-03	2.7E-02	9.1E-01	-2.6E-02	2.5E-02	2.9E-01
rs622568	7	54580201	A	C	2.3E-01	3.7E-02	1.0E-09	3.5E-03	1.4E-02	8.0E-01	5.2E-02	2.6E-02	4.4E-02	-5.7E-02	3.4E-02	9.1E-02	-1.7E-02	3.1E-02	5.8E-01
rs633862	9	133279871	T	C	-1.7E-01	3.4E-02	1.1E-06	-2.6E-02	1.0E-02	1.2E-02	-5.5E-02	1.9E-02	3.8E-03	-5.1E-02	2.4E-02	3.7E-02	-1.0E-04	2.3E-02	1.0E+00
rs6478109	9	114806486	A	G	1.5E-01	2.8E-02	2.4E-07	1.2E-02	1.1E-02	2.5E-01	2.2E-02	2.1E-02	2.8E-01	6.0E-02	2.7E-02	2.7E-02	4.4E-02	2.5E-02	7.4E-02
rs6712600	2	125728622	G	A	-1.7E-01	3.4E-02	3.3E-07	6.9E-03	1.3E-02	5.8E-01	5.0E-02	2.4E-02	3.7E-02	-9.4E-03	3.1E-02	7.6E-01	-3.1E-02	2.9E-02	2.8E-01

rs7135260	12	112943944	T	C	1.9E-01	2.8E-02	6.1E-12	2.6E-02	1.1E-02	1.5E-02	0.0E+00	2.1E-02	1.0E+00	4.0E-02	2.7E-02	1.4E-01	4.1E-02	2.5E-02	1.0E-01
rs77406469	7	150758864	C	T	-3.4E-01	6.9E-02	6.5E-07	1.8E-02	2.7E-02	5.1E-01	-4.3E-02	6.0E-02	4.7E-01	5.4E-02	7.6E-02	4.8E-01	6.6E-02	6.8E-02	3.3E-01
rs77534576	17	49863303	C	T	4.6E-01	7.5E-02	8.5E-10	2.8E-02	3.1E-02	3.7E-01	5.4E-02	6.6E-02	4.1E-01	7.3E-02	8.2E-02	3.7E-01	1.3E-01	7.6E-02	7.6E-02
rs79833209	5	163300447	C	T	4.4E-01	9.2E-02	2.2E-06	1.0E-01	3.6E-02	3.9E-03	8.1E-02	7.9E-02	3.0E-01	-8.4E-03	9.9E-02	9.3E-01	-2.0E-02	9.0E-02	8.2E-01
rs9287218	1	237113798	A	C	-3.2E-01	6.2E-02	3.9E-07	2.8E-03	2.4E-02	9.1E-01	2.3E-02	4.7E-02	6.3E-01	1.2E-02	6.2E-02	8.5E-01	-4.9E-02	5.8E-02	4.0E-01
rs9577175	13	112889041	C	T	2.0E-01	4.1E-02	7.9E-07	-4.0E-03	1.2E-02	7.3E-01	3.8E-03	2.3E-02	8.7E-01	-2.5E-02	3.0E-02	4.0E-01	1.2E-02	2.8E-02	6.7E-01
rs9871880	3	197399535	C	T	-2.5E-01	5.0E-02	4.1E-07	-2.7E-03	1.8E-02	8.8E-01	-1.0E-01	3.6E-02	4.1E-03	5.4E-02	4.4E-02	2.2E-01	1.0E-02	4.2E-02	8.1E-01

Table S4: Sensitivity analysis for the Mendelian randomization analysis of liability to critical Covid-19 on ischemic stroke outcomes including the inverse-variance weighted (IVW) Mendelian randomization and pleiotropy-robust Mendelian randomization approaches (simple, weighted median and MR-Egger). Mendelian randomization estimates represent the odds ratio for ischemic stroke outcomes per unit increase in the log-odds ratio of liability to critical Covid-19. In addition to the Mendelian randomization estimates, we included their 95% confidence interval (CI) and corresponding *p*-value. The intercept of the MR-Egger method was used to test for directional pleiotropy. Instrument selection was based on genetic variants that were associated with liability to critical Covid-19 with a *p*-value equal to or smaller than 5×10^{-6}. Main outcome was any ischemic stroke, and we further included the subtypes cardioembolic stroke, large artery stroke, and small vessel stroke.

Outcome	Method	Estimate	95% CI Lower	95% CI Upper	*p*-value
**Any ischemic stroke**	IVW	1.031	1.004	1.058	0.027
	Simple median	1.022	0.986	1.059	0.227
	Weighted median	1.031	0.995	1.068	0.088
	MR-Egger	1.022	0.947	1.103	0.573
	MR-Egger intercept	0.002	-0.014	0.018	0.820
**Cardio-embolic stroke**	IVW	1.060	1.005	1.119	0.034
	Simple median	1.072	1.001	1.147	0.047
	Weighted median	1.072	1.001	1.147	0.046
	MR-Egger	1.092	0.935	1.276	0.266
	MR-Egger intercept	-0.007	-0.040	0.026	0.689
**Large artery stroke**	IVW	1.067	0.997	1.141	0.059
	Simple median	1.079	0.988	1.180	0.091
	Weighted median	1.087	0.994	1.188	0.066
	MR-Egger	0.919	0.762	1.108	0.374
	MR-Egger intercept	0.034	-0.006	0.073	0.095
**Small vessel stroke**	IVW	1.054	0.999	1.112	0.055
	Simple median	1.075	0.990	1.167	0.087
	Weighted median	1.051	0.968	1.140	0.237
	MR-Egger	1.012	0.867	1.182	0.879
	MR-Egger intercept	0.009	-0.024	0.042	0.585

Table S5: Likelihood ratio test to compare the model fit of the multivariable Mendelian randomization model considering risk for critical Covid-19 as exposure for ischemic stroke outcomes accounting for potential pleiotropic pathways (including life-time smoking index, body mass index, and c-reactive protein) with the univariable Mendelian randomization model. Model fit is evaluated using residual sum of squares for the univariable Mendelian randomization model (RSS 1) with the residual sum of squares for the multivariable Mendelian randomization model (RSS 2). There was one degree of freedom difference between the multivariable and the univariable Mendelian randomization model because there is one additional parameter to estimate in the multivariable Mendelian randomization model. The *F*-statistic quantifies the reduction in residual sum of squares by adding the pleiotropic risk factor to the Mendelian randomization model. The respective *p*-value tests if the multivariable Mendelian randomization model including the pleiotropic pathways provides a significantly better model fit of the genetic association estimates with the stroke outcome than the univariable Mendelian randomization model. Instrument selection was based on genetic variants that were associated with liability to critical Covid-19 with a *p*-value equal to or smaller than 5×10^{-6}. Main outcome was any ischemic stroke, and we further included the subtypes cardioembolic stroke, large artery stroke, and small vessel stroke.

Outcome	Pleiotropic pathway	RSS 1	RSS 2	*F*-statistic	*p*-value
**Any ischemic stroke**	**Smoking**	40.230	39.436	0.584	0.451
	**Body mass index**	40.230	36.919	2.601	0.118
	**C-reactive protein**	37.457	34.841	2.103	0.158
**Cardio-embolic stroke**	**Smoking**	43.899	42.439	0.998	0.326
	**Body mass index**	43.899	43.733	0.110	0.743
	**C-reactive protein**	42.765	37.544	3.894	0.058
**Large artery stroke**	**Smoking**	41.290	40.612	0.483	0.492
	**Body mass index**	41.290	41.290	0.000	0.997
	**C-reactive protein**	40.872	39.648	0.865	0.360
**Small vessel stroke**	**Smoking**	26.742	26.150	0.656	0.425
	**Body mass index**	26.742	23.993	3.322	0.079
	**C-reactive protein**	26.177	26.176	0.001	0.978

Table S6: Mendelian randomization estimates from the inverse-variance weighted Mendelian randomization analysis considering different Covid-19 phenotypes as exposure for ischemic stroke subtypes. Covid-19 phenotypes were based on the definitions by the Covid-19 host genetics initiative. Mendelian randomization estimates represent the odds ratio for ischemic stroke outcomes per unit increase in the log-odds ratio of liability to the respective Covid-19 definition. In addition to the Mendelian randomization estimates, we included their 95% confidence interval (CI) and corresponding *p*-value. Instrument selection was based on genetic variants that were associated with the respective Covid-19 definition with a *p*-value equal to or smaller than 5×10^{-6}. Moreover, we displayed heterogeneity measured by the Q-statistic and the corresponding heterogeneity *p*-value (Het. *p*-value). Main outcome was any ischemic stroke, and we further included the subtypes cardioembolic stroke, large artery stroke, and small vessel stroke.

Exposure	Outcome	Estimate	95% CI Lower	95% CI Upper	*p*-value	Q-statistic	Het. *p*-value
**Hospitalized for Covid-19 *versus* controls with laboratory-confirmed Covid-19**	**Any ischemic stroke**	1.054	1.012	1.099	0.011	5.311	0.915
	**Cardioembolic stroke**	1.044	0.963	1.133	0.294	5.062	0.928
	**Large artery stroke**	1.061	0.957	1.177	0.258	5.080	0.927
	**Small vessel stroke**	1.219	1.107	1.342	5.5×10^{-5}	7.964	0.717
**Hospitalized for Covid-19 *versus* population controls**	**Any ischemic stroke**	1.026	0.981	1.073	0.268	46.406	0.021
	**Cardioembolic stroke**	1.090	0.991	1.198	0.078	56.523	0.002
	**Large artery stroke**	1.081	0.978	1.194	0.128	37.369	0.137
	**Small vessel stroke**	0.991	0.912	1.078	0.841	30.302	0.399
**Reported Covid-19 *versus* population controls**	**Any ischemic stroke**	1.126	1.005	1.262	0.041	59.811	1.1×10^{-4}
	**Cardioembolic stroke**	1.158	0.960	1.396	0.125	44.238	0.010
	**Large artery stroke**	1.464	1.184	1.811	4.2×10^{-4}	33.982	0.108
	**Small vessel stroke**	1.043	0.879	1.237	0.629	25.093	0.457

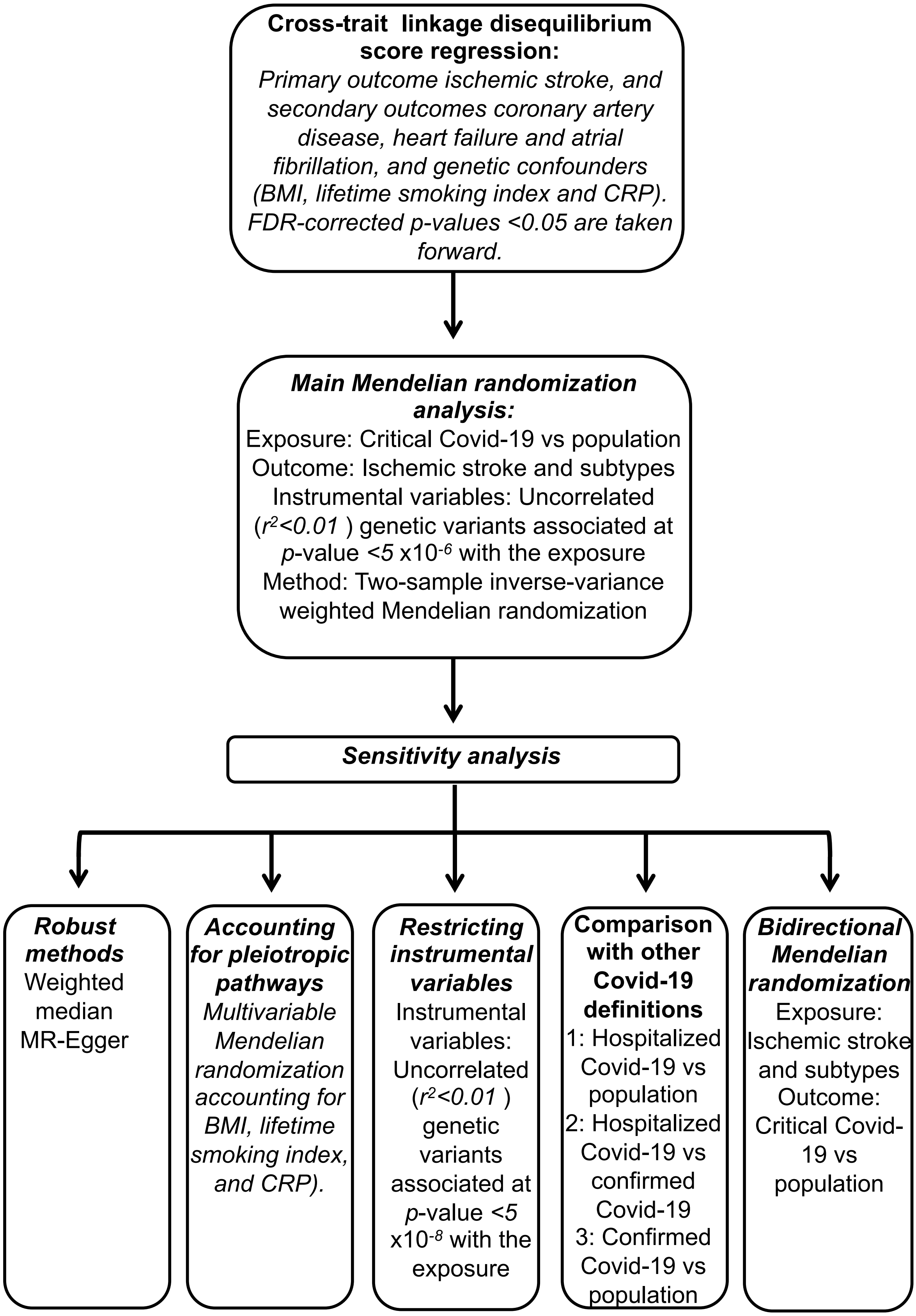

Figure S1: Illustration of the analytical plan.

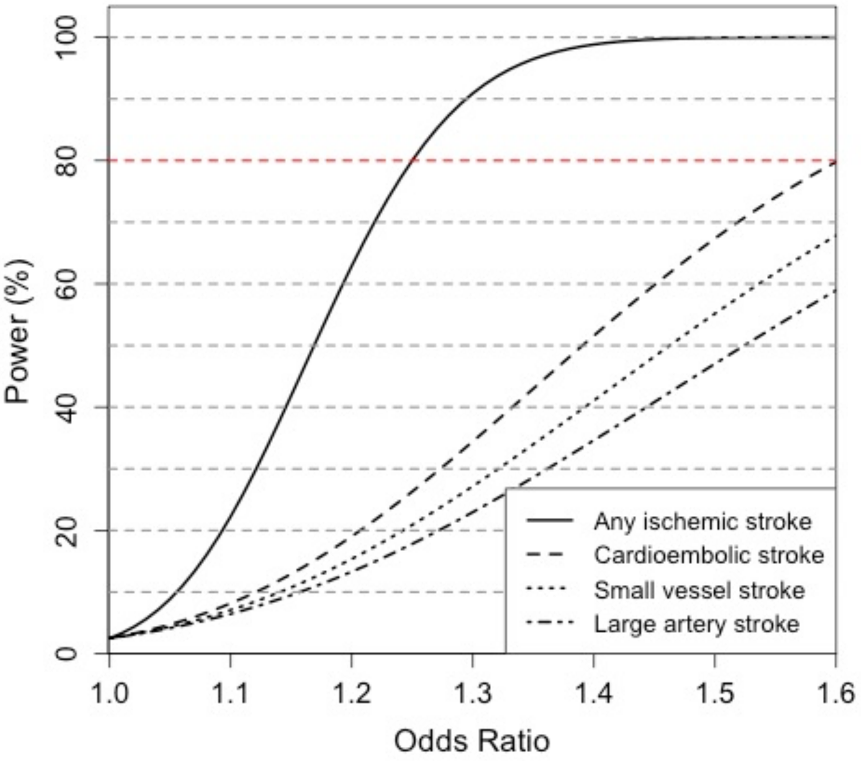

Figure S2: Power calculation showing power (*y*-axis) as a function of Mendelian randomization estimates (odds ratio, x-axis). Different line types describe different case and control numbers representing ischemic stroke and its subtype. See Table S 1 for the exact numbers of cases and control for each outcome. Note that the odds ratio is per one standard deviation unit increase in the exposure, which is not comparable to the Mendelian randomization odds ratios presented in the manuscript which are per unit increase in the log odds ratio of the exposure.

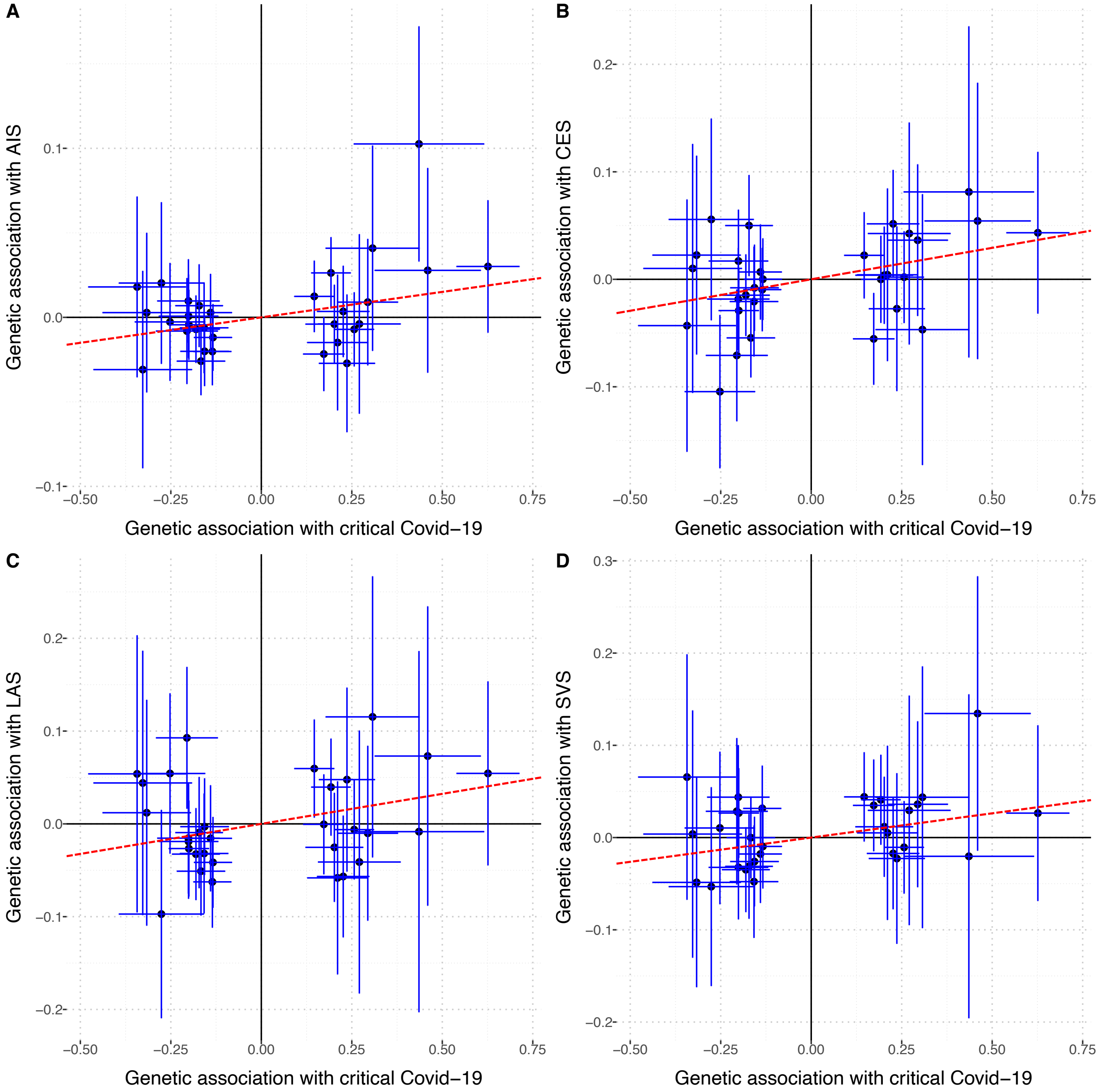

Figure S3: Scatterplots for diagnostics plot the genetic association of the 31 genetic variants used as instrumental variables with the exposure (liability to critical Covid-19) on the *x*-axis against genetic associations with the outcome (ischemic stroke phenotypes) on the *y*-axis. Error bars indicate the standard error of the genetic association. The inverse-variance weighted Mendelian randomization estimate is represented by the red dashed line through the origin. Each panel shows main outcome: Panel **A**) any ischemic stroke (**AIS**), **B**) cardioembolic stroke (**CES**), **C**) large artery stroke (**LAS**), and **D**) small vessel stroke (**SVS**), respectively.

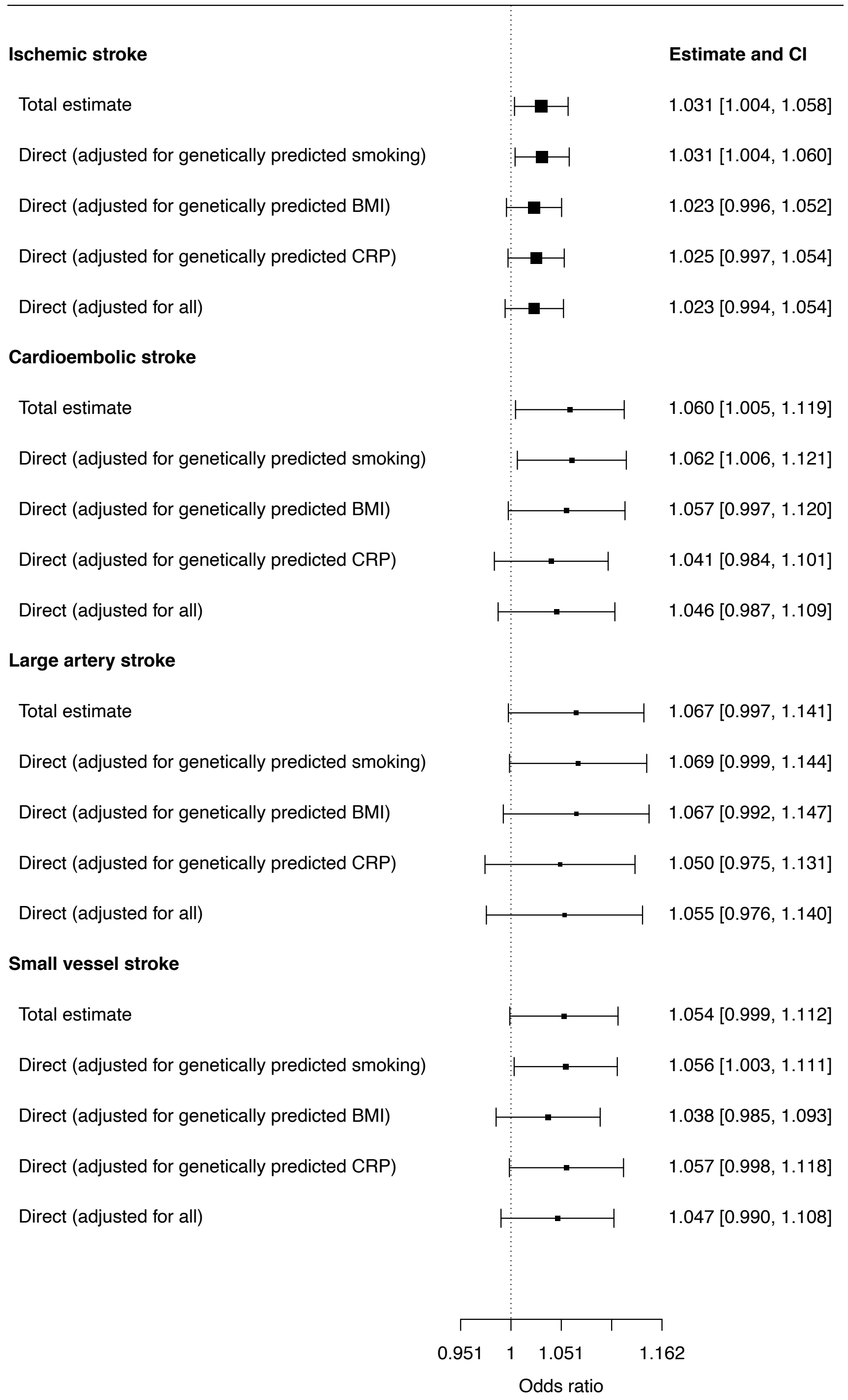

Figure S4: Forest plot contrasting the Mendelian randomization estimates and confidence intervals (CI) from univariable Mendelian randomization (total estimate) and multivariable Mendelian randomization accounting for potential pleiotropic pathways (direct estimate). The total estimate of liability to critical Covid-19 on ischemic stroke outcomes was derived from a univariable (unadjusted) Mendelian randomization model; the direct estimate of Covid-19 on ischemic stroke outcomes was estimated in a multivariable Mendelian randomization model after adjusting for genetically predicted smoking intensity, body mass index (BMI), or C-reactive protein (CRP), and all three potential pleiotropic pathways jointly. Mendelian randomization estimates represent the odds ratio for ischemic stroke outcomes per unit increase in the log-odds ratio of liability to critical Covid-19.

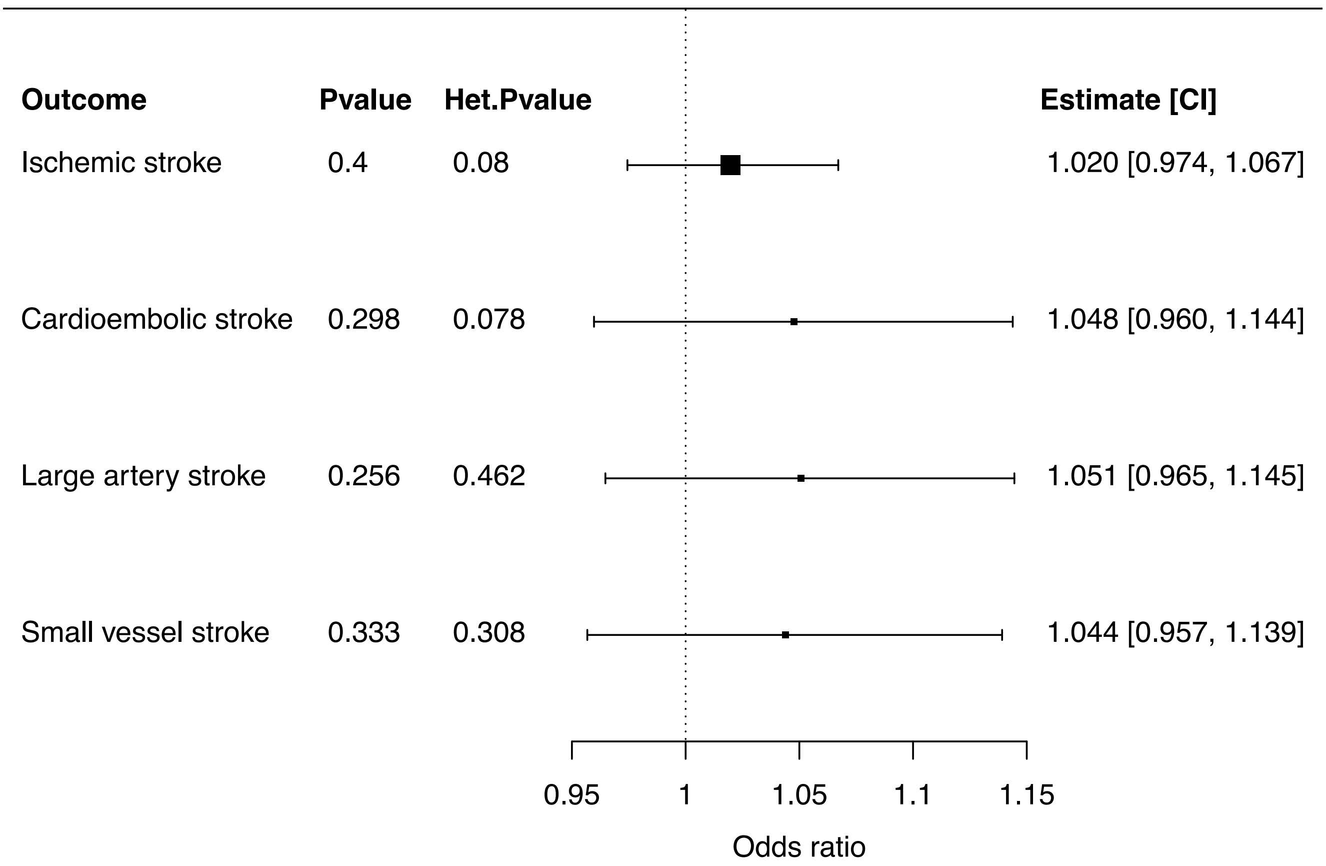

Figure S5: Forest plot illustrating the Mendelian randomization estimates of liability to critical Covid-19 on stroke outcomes based on inverse-variance weighted Mendelian randomization using 9 genetic variants which were associated with liability to critical Covid-19 at genome-wide significance (*p*-value $< 5\times10^{-8}$). Mendelian randomization estimates represent the odds ratio for ischemic stroke outcomes per unit increase in the log-odds ratio of critical Covid-19 liability. Additional columns include the *p*-value (*p*-value) of the Mendelian randomization estimate to be different from the null, represented by a dashed line at an odds ratio of 1, and the heterogeneity of the Mendelian randomization model measured by the heterogeneity *p*-value (Het. *p*-value) as well as the Mendelian randomization estimate and its 95% confidence interval (CI).

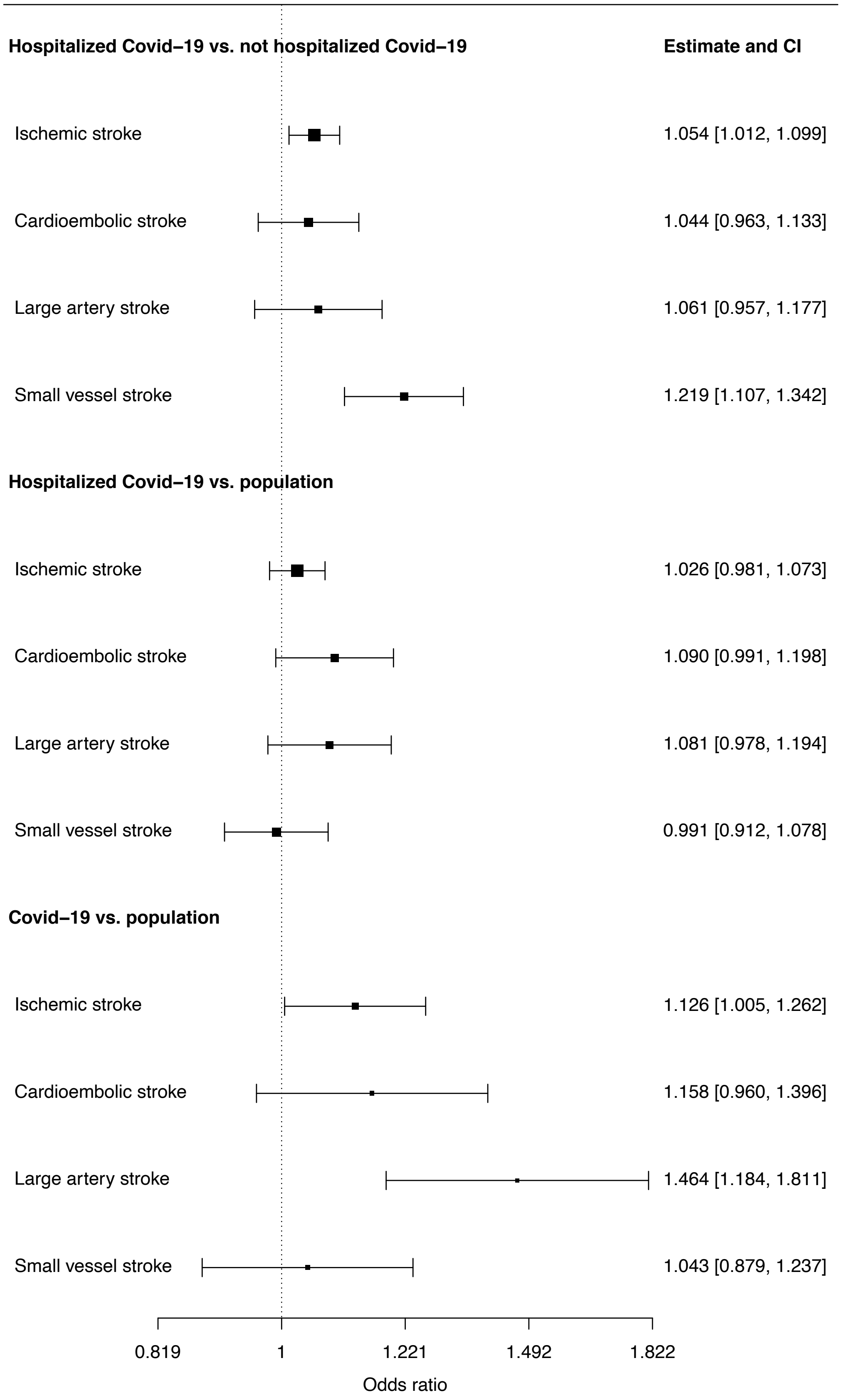

Figure S6: Forest plot illustrating the inverse-variance weighted Mendelian randomization estimate and 95% confidence interval (CI) considering Covid-19 phenotypes as exposure for ischemic stroke outcomes. Covid-19 phenotypes were based on the definitions by the Covid-19 host genetics initiative. Mendelian randomization estimates represent the odds ratio for ischemic stroke outcomes per unit increase in the log-odds ratio of liability to the respective Covid-19 definition. Genetic variants which were associated with the Covid-19 definition were selected as instrumental variables at a *p*-value level equal to 5×10^{-6} or smaller.
